# Supplementary material for: A multipath error cancellation method based on antenna jitter
Source: Commun Eng. 2025 Feb 7;4:17. doi: 10.1038/s44172-025-00355-z (PMC11806055; doi:10.1038/s44172-025-00355-z)
Supplement: Supplementary file 2 — Supplementary Information [file 44172_2025_355_MOESM2_ESM.pdf]

Communications Engineering

A Multipath Error Cancellation Method Based on Antenna Jitter

(Supplementary Information)

Contents

Supplementary Note 1—The detailed specifications of the monitoring antenna and receiver used in the experiment. 3
Supplementary Note 2—The information on commonly available commercial vehicle-mounted antennas was selected for comparison in this experiment. 4
Supplementary Fig. 1 Statistical data of multipath error in the complex platform. 4
Supplementary Fig. 2 Relationship between relative phase and multipath error. The diagram illustrates a specific correlator spacing, which is used solely to demonstrate the impact of multipath signals. Any traditional correlator spacing is applicable to the method proposed in this paper. 5
Supplementary Fig. 2 Relationship between antenna jitter and multipath error. 6
Supplementary Fig. 3 Bias of Direct signal estimation. 7
Supplementary Fig. 4 The variation trend of multipath error versus jitter. 8
Supplementary Fig. 5 The variation trend of multipath error versus jitter amplitude under different time delays. 9
Supplementary Fig. 6 The envelope of the 1-chip correlation distance. 10
Supplementary Fig. 7 Multipath error envelopes with different correlation distances. 11
Supplementary Fig. 8 The variation trend of multipath error versus the number of reflective surfaces under different antenna states. 12
Supplementary Fig. 9 The variation trend of multipath error versus the number of reflective surfaces under no main reflective surface. 13
Supplementary Fig. 10 Comparison of Multipath Mitigation Algorithms 14
Supplementary Table 1 Parameters of reflective surface. 15
Supplementary Table 2 Parameters of multiple reflective surfaces. 16
Supplementary Table 3 Parameters of multiple reflective surfaces (no main reflective surface). 17

Supplementary Note 1——The detailed specifications of the monitoring antenna and receiver used in the experiment.

Both components were independently developed by our institution as follows:

Monitoring antenna Monitoring receiver

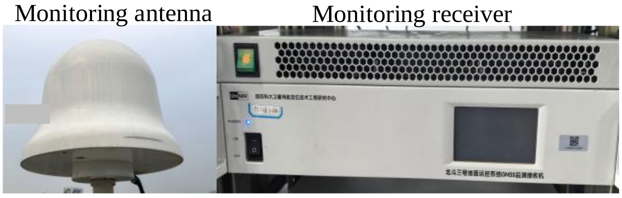

The performance parameters related to this experiment are as follows:

Monitoring antenna parameters:

Parameters	Values
Gain	≥40dB
Support system	BD/GPS/GLONASS/Galileo
Polarization method	Right-circular polarization
Choke ring	Yes
Voltage	DC 5V
Impedance	50 Ω

Monitoring receiver parameters:

Parameters	Values
Pseudorange accuracy	10 cm
BDS	B1, B3
GPS	L1, L2
GLONASS	L1, L2
Galileo	E1 E5b
Time to first fix	Hot(<15s) Cold(<60s)

Supplementary Note 2—The information on the commonly available commercial vehicle-mounted antennas was selected for comparison in this experiment.

A visual comparison between these antennas and the monitoring antenna is presented as follows:

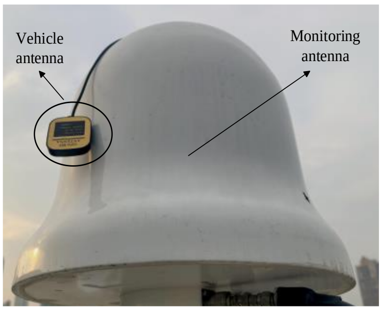

The performance parameters related to this experiment are listed as follows:

Parameters	Values
Gain	28±2 dB
Support system	BD/GPS
Polarization method	Right-circular polarization
Choke ring	No
Voltage	DC 3-5V
Impedance	50 Ω

Supplementary Figures

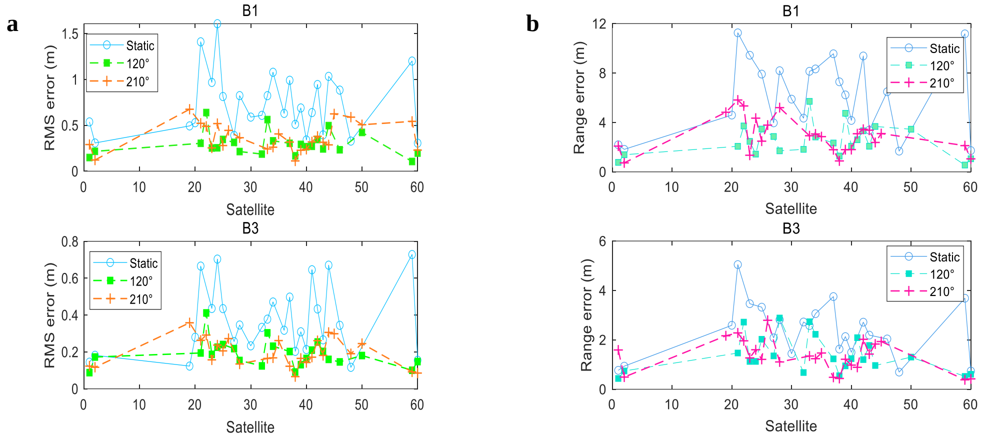

Supplementary Fig. 1 Statistical data of multipath error in the complex platform. (a) A notable observation from Supplementary Fig. 1 is that the antenna jitter exhibits a more pronounced multipath error reduction in complex platforms than in open platforms. As the complexity of the experimental environment escalates, there is an obvious increase in the MS error for static antennas. In contrast, the MS error for jitter antennas (120 or 210°) displays a far less pronounced change. Furthermore, Supplementary Fig. 1 illustrates a diminished difference in the jitter direction between 120° and 210° (represented by the red and green lines). This suggests that in complex environments, the sensitivity to the direction of jitter is lessened, indicating that antenna dithering is more adaptable to varying environmental conditions. To enable a direct comparison with the open platform, Table 3 presents a comprehensive statistical analysis for satellites No. 1 and No. 59. (b) Antenna jitter has an additional eliminating effect on the MEO satellites. Although the multipath error of MEO satellites is inherently dynamic, it requires tens of minutes of sampling to eliminate by averaging. What stands out is that antenna jitter can reduce that time to seconds. Specifically, the MS error of MEO satellites can be decreased from 0.89m to 0.37m, which demonstrates the substantial impact of antenna jitter on mitigating multipath errors across various satellite types.

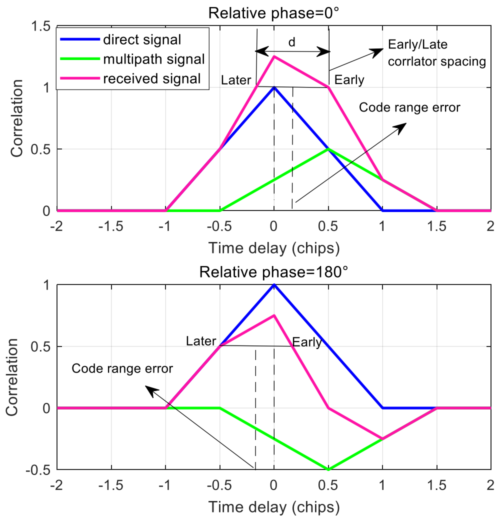

Supplementary Fig. 2 Relationship between relative phase and multipath error. The diagram illustrates a specific correlator spacing, which is used solely to demonstrate the impact of multipath signals. Any traditional correlator spacing is applicable to the method proposed in this paper. Supplementary Fig. 2 illustrates the GNSS signal autocorrelation function in the two cases of different relative phases, where the multipath amplitude ratio a_1 is 0.5, the E-L spacing $d = 0.7$ chip, and the multipath delay $\Delta\tau_1$ is 0.5 chip. As shown in Supplementary Fig. 2, it is apparent that the relative phase of the multipath signal is in-phase or anti-phase, and the maximum multipath error is obtained, i.e. $\varepsilon_{\max} = \pm a_1 d / 2$. Furthermore, it should be noted that the error is positive when the relative phase ranges from 0 to 90 degrees, and negative when the relative phase ranges from 90 to 180 degrees, which allows us to randomize the multipath error by manipulating the relative phase.

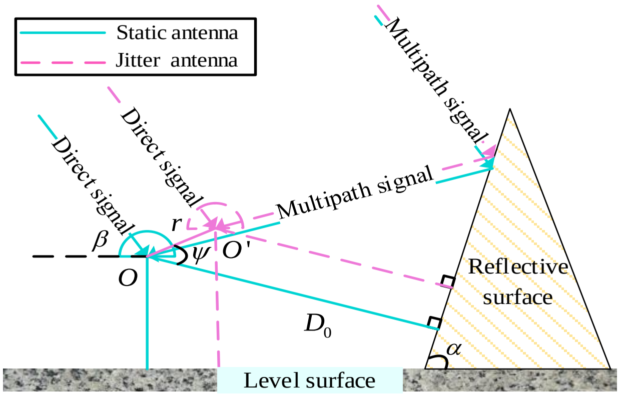

Supplementary Fig. 3 Relationship between antenna jitter and multipath error. As shown in Supplementary Fig. 3, α is the angle of the reflective surface relative to the horizontal surface, where various angles of reflection correspond to different scenarios (ground, buildings, slopes, etc.), and β is the elevation angle of the satellite. The perpendicular distance from the antenna phase center O to the reflective surface is D_0 . Then, we establish a polar coordinate system making O as the origin and the vertical line from O to the reflecting surface as the polar axis. Hence, the position of the jitter antenna phase center O' can be determined by the distance r_n from O' to O and the angle ψ_n between $O'O$ and the polar axis at the sample moment n .

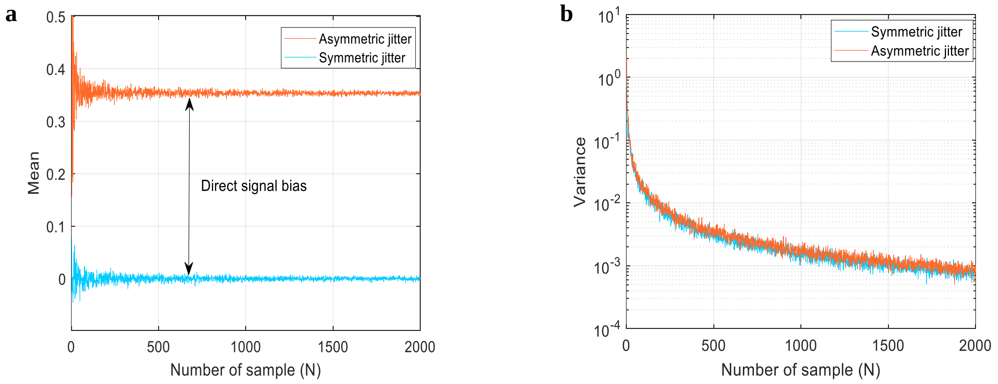

Supplementary Fig. 4 Bias of Direct signal estimation (a) Mean, (b) Variance. Supplementary Fig. 4 (a) and (b) show the convergence of the mean and variance for symmetric and asymmetric jitter, respectively. It can be observed in Supplementary Fig. 4 that bias is fully negated upon averaging with symmetric jitter. Conversely, the bias post-averaging remains unmitigated with asymmetric jitter. Thus, adjustments based on the jitter trajectory are required when symmetric jitter cannot be implemented, yet achieving precise corrections is notably difficult. On the other hand, the variance of the direct signal bias decreases progressively as the number of samples increases. Once the number of samples exceeds 1000, the variance reduction exhibits a linear trend and below 10^{-5} m, meeting the requirements for real-time high-precision positioning.

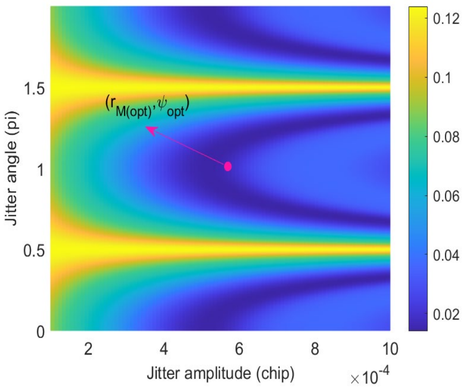

Supplementary Fig. 5 The variation trend of multipath error versus jitter. Supplementary Fig. 5 illustrates the variation trend of multipath error versus jitter amplitude and angle. Here, the multipath delay $\Delta\tau_1$ is 0.01 chip, the multipath power ratio a_1 is 0.5, the satellite elevation angle β is 30°, the reflective surface inclination α is 90°, and the carrier frequency is 1561.98 MHz. It can be seen from the comparison in Supplementary Fig. 5 that the multipath error decreases different values with the various jitter amplitude and angle, where the jitter angle and amplitude of minimum error are strongly consistent with the theoretical analysis. The above jitter angle and amplitude are equivalent to the antenna jittering ±5cm along the direction normal to the reflecting surface, such a jitter amplitude is acceptable in the real environment. Additionally, it should be noted that multipath error diverges when jitter angles are $\pi / 2$ and $3\pi / 2$, i.e., the jitter directions are parallel to the reflecting surface. This also confirms the derivation in Theorem 1, which emphasizes the need to select a reasonable angle.

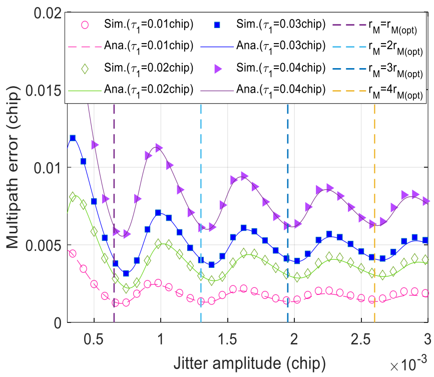

Supplementary Fig. 6 The variation trend of multipath error versus jitter amplitude under different time delays. Supplementary Fig. 6 illustrates the variation trend of multipath error versus jitter amplitude for different time delays, where "Sim" refers to the simulated results obtained through numerical simulations, while "Ana" refers to the analytical results, which are derived from the theoretical analysis in the paper. As can be seen from Supplementary Fig. 6, it is evident that the multipath error does not monotonically decrease, as the jitter amplitude increases. Instead, it reaches different minimum values at $k = 1, 2, 3,$ and 4 , which validates the accuracy of Theorem 2 and Theorem 3 under conditions of short time delay. However, multiple multipath signals with diverse time delays are encountered in practical scenarios. Thus, assessing the efficacy of antenna jitter in reducing multipath errors necessitates consideration of all time delays, typically represented using multipath error envelopes. Supplementary Fig. 7 illustrates the multipath error envelopes of the 1-chip correlation distance.

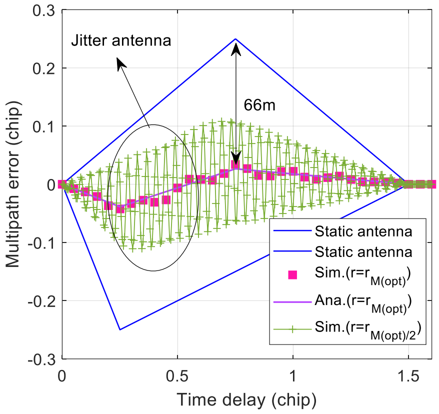

Supplementary Fig. 7 The envelope of the 1-chip correlation distance. Supplementary Fig. 7 illustrates the multipath error envelopes of the 1-chip correlation distance. From Supplementary Fig. 7 we can see that the jitter antenna exhibits an obvious suppression effect on multipath errors across all time delays, where the peak value and effective area of the multipath error envelope are notably reduced. Specifically, the peak error can be reduced by 66% when employing half of the optimal jitter amplitude, and the peak error has been reduced by 66 m compared to the static antenna when employing the optimal jitter amplitude. Furthermore, the theoretical and simulation results are in excellent agreement, which comprehensively verifies the correctness of Theorem 3. To verify the combined performance of the methods presented in this paper with other approaches.

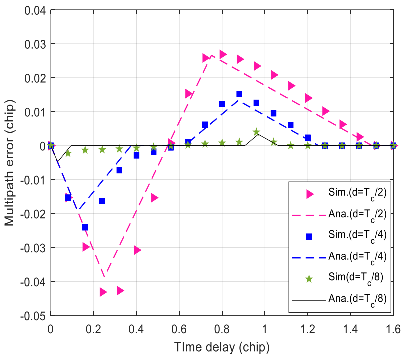

Supplementary Fig. 8 Multipath error envelopes with different correlation distances. Supplementary Fig. 8 validates the case when the antenna jitter is combined with narrow correlation technology. In supplementary Fig. 8, there is a clear downward trend of multipath when combined with narrow correlation technology. In particular, the envelope peak value of the correlation distance of the 1/8 chip can be further reduced by 4/5, and the effective length of the envelope can be reduced by 7/8 compared to the correlation distance of the 1/2 chip. As a result, the envelope peak value can be reduced by 98%, equivalent to around 0.005 chips. Moreover, approximately 87.5% of the overall time delay of the multipath error can be minimized to approach zero when random errors in the pseudorange measurements are not considered, indicating the theoretical effectiveness of the antenna jitter.

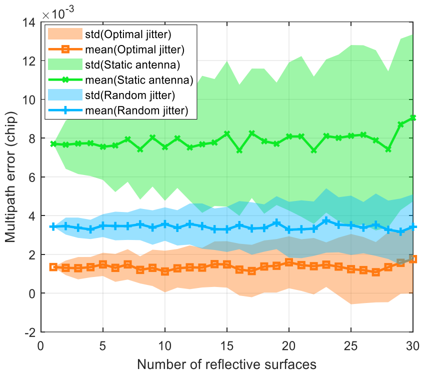

Supplementary Fig. 9 The variation trend of multipath error versus the number of reflective surfaces under different antenna states. Supplementary Fig. 9 presents the mean and mean square (MS) deviation across varying counts of reflective surfaces. The comparison of Supplementary Fig. 9 reveals that the optimal jitter mode, previously determined for a single reflective surface, remains effective even when multiple reflective surfaces are present. Specifically, the MS error associated with the jitter antenna is markedly lower than the static antenna, and this discrepancy tends to increase with the addition of reflective surfaces. Furthermore, the optimal jitter method yields markedly smaller multipath errors than other antenna states, because it accounts for the calibration of the primary reflective surface. This suggests that the optimal jitter direction and angle play an important role in real scenarios if the primary reflective surface can be calibrated.

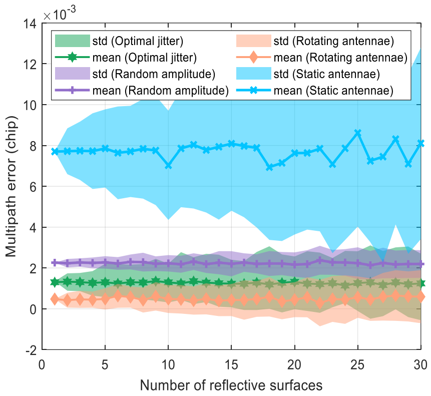

Supplementary Fig. 10 The variation trend of multipath error versus the number of reflective surfaces under no main reflective surface. Supplementary Fig. 10 compares the performance of this calibrated setup with that of a rotating antenna at the same amplitude, as well as with a system that employs a random jitter amplitude but the same direction. It can be observed that an increase in jitter amplitude primarily results in a reduction of variance within scenarios featuring a higher number of reflective surfaces in Supplementary Fig. 10. Conversely, the mean value of the multipath error tends to increase, which underscores the efficacy of an optimally determined jitter amplitude. Furthermore, the discrepancy between the multipath errors generated by the optimal jitter method and those produced by a rotating antenna is found to be minimal. This finding validates that linear jitter is equally effective as the rotating antenna technique on multiple reflective surfaces, with the added benefit of conserving space.

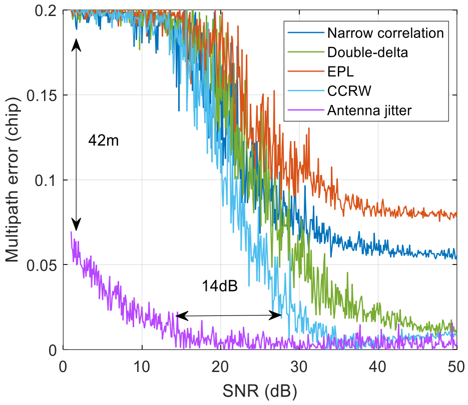

Supplementary Fig. 11 Comparison of Multipath Mitigation Algorithms. As shown in Supplementary Fig. 11, it is evident that the antenna jitter possesses distinct advantages in terms of robustness and efficacy. This technique demonstrates remarkable performance under low SNR conditions, achieving steady-state convergence 10-15 dB earlier than other methods, which is particularly suited to counteract the complexities of urban and other challenging environments. Furthermore, the error during the steady state of this method is reduced by approximately 80%-90% compared to traditional narrow correlation and Early-Prompt-Late (EPL) methods, even surpassing the performance of more complex algorithms such as Code Correlation Reference Waveform (CCRW) 13 and Double-delta, which is attributed to the introduction of observables through motion. Thus, this method enables the direct application to conventional receivers as an independent parameter. This results in low-complexity, high-precision positioning and it can be integrated with other high-precision algorithms to meet the ultra-high-precision requirements of monitoring stations.

Supplementary Tables

Supplementary Table 1 Parameters of reflective surface.

Parameters	Values
Angle (reflective surfaces)	90°
Angle (satellite elevation angle)	45°
maximum amplitude	5 cm
jitter angle	0° /180°

Supplementary Table 2 Parameters of multiple reflective surfaces.

Parameters	Values
Angle (main reflective surface)	90°
Relative delay (main reflective surface)	0.01 chips
Power ratio (main reflective surface)	0.5
Angle (other reflective surfaces)	[80°, 135°]
Relative delay (other reflective surfaces)	[0, 2] chips
Power ratio (other reflective surfaces)	[0, 0.3]

Supplementary Table 3 Parameters of multiple reflective surfaces (no main reflective surface).

Parameters	Values
Angle (reflective surfaces)	[80°, 135°]
Relative delay (reflective surfaces)	[0, 2] chips
Power ratio (reflective surfaces)	[0, 0.3]
Radius of rotation	$r_{M(opt)}$
Random amplitude	$[1,2]\times r_{M(opt)}$
